# The magnitude of gender-based violence, health consequences, and associated factors among women living in post-war woredas of North Shewa zone, Amhara, Ethiopia, 2022

**DOI:** 10.3389/fgwh.2024.1335254

**Published:** 2024-05-07

**Authors:** Eyosiyas Yeshialem Asefa, Assalif Beyene Haile, Osman Yimer Mohamed, Dagimawit Berhanu

**Affiliations:** ^1^ School of Public Health, Asrat Woldeyes Health Science Campus, Debre Berhan University, Debre Berhan, Ethiopia; ^2^ Department of Midwifery, School of Nursing & Midwifery, Asrat Woldeyes Health Science Campus, Debre Berhan University, Debre Berhan, Ethiopia; ^3^ Department of Nursing, School of Nursing & Midwifery, Asrat Woldeyes Health Science Campus, Debre Berhan University, Debre Berhan, Ethiopia

**Keywords:** abortion, anxiety, gender-based violence, sexual partner, school dropout

## Abstract

**Background:**

Gender-based violence (GBV) is a pervasive global public health concern and a violation of human rights, particularly pronounced in conflict settings where it is often used as a tool of warfare to instill fear and control populations.

**Objective:**

Assessment of Magnitude, Associated Factors, and Health Consequences of GBV among women living in war-affected woredas of North Shewa zone, Ethiopia, 2022

**Methods:**

A community-based cross-sectional study was conducted, involving 845 randomly selected women living in conflict zones. Data on GBV experiences over the previous 3 months were collected through interviewer-administered questionnaires developed from literature review. The collected data underwent validation, entry into EPI data, and analysis using SPSS. Findings are summarized using descriptive statistics, AOR and 95% confidence interval.

**Result:**

The magnitude of GBV in this study was (490, 58.0%) where, (466, 55.0%) psychological violence, (254, 30.1%) physical violence, and (135, 16.0%) reported sexual violence. A majority of the physical violence, (161, 63.4%), occurred during conflict period, with (143, 56.3%) of these cases involving armed forces, and (161, 63.4%) women experiencing physical violence in their homes. Urban Residence AOR = 2.65, CI, (1.82–3.89), Educational status of Secondary education AOR = 0.33, CI, (0.19–0.57, and ≥College AOR = 0.17, CI, (0.09–0.35), Occupation of Housewife AOR = 1.88, CI, (1.20–2.94), Private employee AOR = 6.95, CI, (3.70–13.04), Gov't employee AOR = 5.80, CI, (2.92–11.50), and others (Students) AOR = 3.46, CI, (1.98–6.01), Ever had sexual intercourse AOR = 0.46, CI, (0.25–0.83), Have heard about SRH AOR = 0.59, CI, (0.40–0.89), Have had previous GBV exposure AOR = 0.24, CI, (0.15–0.38), having a previous history of sexual violence AOR = 0.30, CI, (0.16–0.57), and Number of sexual partner AOR = 1.84, CI, (1.13–2.99) were identified to be associated factors of GBV in our study area. The most commonly reported consequences of GBV were Anxiety, depression, physical injuries, self-blame, women had school dropout, and abortion.

**Conclusion:**

The study reveals a higher prevalence of GBV, resulting in profound physical, social, mental, and reproductive health challenges for survivors. To address this, multi-sectoral cooperation is advised to enhance women's empowerment, access to information, and psycho-social support in affected areas. Furthermore, national policymakers are urged to implement preventive measures during conflict and establish legal mechanisms to ensure accountability for perpetrators.

## Introduction

GBV represents a worldwide public health concern and a breach of human rights. As broadly outlined by the United Nations, GBV encompasses any form of violent behavior that is committed due to socially attributed gender distinctions. This inclusive categorization includes various forms of intimate partner violence (IPV) and sexual assault not involving a partner, as well as a spectrum of aggressive actions, including physical, psychological, economic, and sexual violence, exploitative or coercive behaviors, and detrimental traditional customs (1).

GBV is described as violence targeting individuals based on their sex or gender, resulting in psychological, physical, or sexual trauma, whether directly or indirectly (2). GBV represents one of the most prevalent, yet underreported, and unprovoked human rights violations globally.

Over the last decade, extensive documentation has shed light on the issue of GBV in conflict zones and its devastating impact on the lives of those affected by conflicts (3, 4). GBV has been recognized as a weapon in times of war or conflict, often used as a tool for controlling and instilling fear in populations. However, it is a highly diverse phenomenon in terms of its occurrence and those responsible for it (5). Much of the research has focused on violations against women and girls, including instances of rape during wartime and various forms of violence, such as child sexual abuse, forced or coerced prostitution, human trafficking, and other forms of sexual exploitation (6).

GBV can also be defined as violence directed at minority groups, individuals, or communities solely based on their gender, which can result in psychological, physical, and sexual trauma, as well as the denial of their fundamental human rights (6).

Data from the World Health Organization (WHO) and its partners indicate that violence against women remains alarmingly widespread and commences at a young age. Approximately 1 in 3 women, or around 736 million women, experience physical or sexual violence from an intimate partner or sexual violence from a non-partner during their lifetime. Shockingly, this number has remained largely unchanged over the past decade. The violence often begins at a young age, with 1 in 4 young women (aged 15–24) experiencing violence from an intimate partner by the time they reach their mid-twenties. Intimate partner violence is the most prevalent form of violence against women globally, affecting approximately 641 million women. Additionally, 6% of women worldwide report being sexually assaulted by someone other than their husband or partner. Due to the significant levels of stigma and underreporting of sexual abuse, the actual figure is likely much higher (7). A systematic review based on more than 50 population-based surveys worldwide estimates that between 8% and 70% of women have experienced sexual assault by a male partner at least once in their lifetime, and about 10%–50% of women have been physically beaten by their intimate partners (8).

The situation in northern Ethiopia has remained highly unpredictable and volatile, marked by ongoing fighting in multiple areas and dynamic front lines that significantly affect humanitarian access and response, putting civilians at great risk. The conflict has led to increased humanitarian needs, primarily due to large-scale displacements. By the end of July, over 2.1 million people had been displaced across 695 sites in the Tigray, Amhara, and Afar regions, according to the International Organization for Migration (9).

Ethiopia is a signatory to international agreements promoting gender equality and the rights of women and girls, including the Convention on the Elimination of all Forms of Discrimination against Women (CEDAW), the Beijing Declaration and Platform for Action (BDPFA), and the Protocol to the African Charter on the Rights of Women in Africa (Maputo Protocol) (10).

The conflict erupted in November 2020 between Tigrayan armed forces and Ethiopian defense forces in the Tigray region and subsequently expanded to neighboring areas in the Afar and Amhara regions. Armed Tigrayan forces invaded many woredas in the North Shewa zone of the Amhara region in 2022 for approximately 2 months. Such conflicts typically weaken social stability and expose vulnerable population groups, such as women and children, to various forms of violence (11–13).

Unlike other regions of the world, there is no available data regarding the extent of GBV, its health consequences, and associated factors in the study area even before the outbreak of war. Therefore, this study aims to assess the magnitude, associated factors, and health consequences of GBV among women living in post-war woredas of the North Shewa zone, Amhara, Ethiopia in 2022.

## Methods

### Study area and period

This study was carried out in the woredas of North Shewa Zone, which have been affected by a conflict between government forces and Tigrayan armed forces. The North Shewa Zone is one of the ten Zones located within the Amhara Region of Ethiopia. Its name is derived from the historical kingdom or former province of Shewa. This Zone is bordered by the Oromia Region to the south and west, South Wollo to the north, the Oromia Zone to the northeast, and the Afar Region to the east. The North Shewa Zone consists of 24 administrative woredas.

During the course of the conflict, various woredas within North Shewa Zone, including Menz Gera Midir, Menz Lalo Midir, Menz Mama Midir, Menz Keya Midir, Geshe Rabe, Efratana Gidim, Kewot, Antsokiyana Gemza, Mojana Wadera, and Tarmaber, were invaded by the Tigray armed force and Shene armed groups for varying durations. The study took place in four woredas chosen randomly from the ones mentioned above and data collection period was from 10 April to 15, 2022.

### Study design

The community-based cross-sectional study design was applied.

### Source and study population

#### Source population

All women residing in war-affected woredas of north Shewa Zone.

#### Study population

Randomly selected women from selected households residing in post-war woredas of the North Shewa zone.

#### Inclusion and exclusion criteria

##### Inclusion criteria

Women who were available in the selected households of post-war woredas of the North Shewa zone were included.

##### Exclusion criteria

Women who were critically ill and unable to communicate and those who were displaced to uninvaded areas during wartime were excluded.

### Sample size determination

The sample size for this study was determined using single population proportion formula with considering the following assumptions:

P = Proportion of women who ever experienced physical, sexual, and/or psychological abuse in Gondar, Northwest Ethiopia, was 50.8%, (*p* = 0.508) (14),

*Z α*/2 = critical value for normal distribution at 95% confidence level which equals 1.96 (*Z*-value at alpha = 0.05). The margin of error is to be 5% (*d* = 0.05).

$$n=\;\frac{{\left(Z\frac{\alpha }{2}\right)}^{2}P(1-P)}{d2}=384$$

Considering the design effect of 2% and 10% of the non-response rate the minimum final sample size becomes 845.

### Sampling technique

In the selected woredas, a representative portion of the kebeles (30%) were chosen using a simple random sampling technique. Subsequently, a systematic random sampling approach was employed to select households, with all households in the selected kebeles being assigned numbers for this purpose. The final sample consisted of 845 women, selected based on calculated interval K, which was determined by dividing the total number of households in the selected kebeles by the proportional sample size.

### Data collection tools and procedures

The interviewer administered questionnaire in the Amharic language, which had been translated from the original English version was used for data collection. To ensure the validity of our tool, a pretest was conducted on 5% of the samples, involving 42 participants, in the non-selected woreda. Additionally, we assessed the reliability of our tool, and the Cronbach's Alpha value obtained was 0.83, indicating good reliability.

The data collection process involved four supervisors, each holding a master's degree in health science, and 20 trained data collectors, including BSc nurses and midwives. A one-day training session was organized for the data collectors and supervisors. This training covered various aspects, such as how to conduct data collection, an explanation of the study's objectives, and guidance on providing psychological support to Survivors and referring them for further assistance.

### Study variable

#### Dependent/outcome variable

GBV (psychological, physical, sexual) and Health consequences.

#### Independent variables

Socio-Demographic and Economic Variables (Age, residence, Sex, Religion, Ethnicity, Marital Status, living condition, Educational Status, occupation, monthly income), Sexual and reproductive characteristics (Ever had sexual intercourse, Age at first sex, Number of the sexual partner in life, How you were exposed to your first sexual intercourse, Discussion SRH issues with family and or friends, Having constant sexual partners, previous GBV exposure), Previous fertility characteristics (ever been pregnant, ever have an unwanted pregnancy, ever had an abortion, ever had induced abortion, parity, Number of alive children), Healthcare-related factors (Availability of health facility, getting immediate service), and Conflict settings: Types of Perpetrators: (Armed actors, intimate partners, family members), location (In community during the conflict, Home), Number of perpetrators.

### Operational definition

GBV is defined as violence directed against an individual based on sex or gender, which includes.

#### Physical violence

Scratching, pushing, shoving, throwing, grabbing, biting, choking, shaking, poking, hair-pulling, slapping, punching, hitting, burning, or the use of a weapon such as a gun, knife, or other objects. If a study participant experienced any of the actions listed above, she was categorized as a survivor of physical violence.

#### Sexual violence

The use of physical force to coerce an individual into participating in a sexual act against their will, regardless of whether the act is completed. An attempted or completed sexual act involving a person who is incapable of comprehending the nature or circumstances of the act, unable to refuse participation, or unable to communicate their unwillingness to engage in the sexual act. Additionally, abusive sexual contact, which involves intentionally touching the genitalia, anus, groin, breast, inner thigh, or buttocks of any individual against their will, or any person. If a study participant experienced any of the situations mentioned above, she was classified as a survivor of Sexual Violence.

#### Psychological/emotional violence

Emotional harm experienced by the survivor due to actions, threats of actions, or coercive strategies, especially when preceded by prior instances of physical or sexual violence or previous threats of physical or sexual violence, such as intimidation, continual demeaning, and humiliation. If a study participant encountered any of the situations mentioned above, she was categorized as a survivor of psychological violence.

Generally, If the study participant experienced any of the aforementioned forms of violence (physical, sexual, or psychological violence), she was identified as a survivor of GBV (GBV).

#### Health consequences

Include physical injuries, sexual health issues, reproductive health complications, psychological trauma, substance abuse, chronic health conditions, interpersonal relationship issues, economic consequences, violations of reproductive rights, and impacts on child health.

### Data quality control

The instrument underwent a pretest on a 5% sample in non-selected woredas. After the pre-test, essential modifications were made to improve the tool's efficiency before initiating the actual data collection process. Thorough training was conducted for both the data collectors and supervisors. Supervisors conducted daily reviews and checks of the questionnaires to ensure their completeness and relevance. The tools' reliability and convergent validity were also evaluated.

### Data processing and analysis

After collecting the data, each questionnaire underwent a visual check for completeness, manual cleaning, coding, and entry into Epi Data 3.1. Subsequently, the information was transferred to SPSS version 25 software for subsequent analysis. The normality of data distribution was also checked, and data adjustments were made when needed.

To examine the relationship between dependent and independent variables, multivariable logistic regression analysis was utilized, and the prerequisites for multiple variable logistic regression were confirmed. Variables exhibiting a *p*-value lower than 0.2 in the bivariate logistic regression were chosen for inclusion in the multiple variable logistic regression analysis. The existence and magnitude of associations were established through odds ratios, 95% confidence intervals (CI), and the corresponding *p*-values. Ultimately, the findings were summarized and presented through statements, tables, and graphs, utilizing adjusted odds ratios and their respective 95% CIs.

### Ethical consideration

We obtained ethical clearance from the Institutional Review Board (IRB) at the Asrat Woldeyes Health Sciences campus and formally notified the Woreda and Kebele administrations. Every research participant submitted written informed consent, and for women below the age of 18, an assent process was also conducted. This included using a specially prepared assent form along with guardian consent.

In cases where individuals were unable to read or write, a witness ensured that the consent information was accurately explained, that the subject fully understood the details, and that informed consent was given voluntarily.

Throughout the research process, participants were afforded the right to decide on their participation. To protect confidentiality, the names of participants were omitted from the questionnaire. The data collected were handled with the utmost confidentiality and used exclusively for the study's purposes. This study was conducted in accordance with the principles outlined in the Declaration of Helsinki.

## Results

### Socio-demographic characteristics of the respondents

The study included a total of 845 respondents, resulting in a response rate of 100%. The participants' ages ranged from 16 to 80, with a mean age of 29.25 years and a standard deviation of ±8.97. Three-quarters (634, 75.0%) of the study participants resided in rural areas, and a significant majority (727, 86.0%) identified as Orthodox religious followers.

More than half of the respondents (470, 55.6%) were currently married, and (427, 50.5%) of the women were living with their husbands or friends. Among the total respondents, (455, 53.80%) had families consisting of 3–5 members, while (296, 35.0%) reported a monthly income of less than 36 USD (Table 1).

**Table 1:** Socio-demographic characteristics of respondents, 2022.

Socio-demographic characteristics	Count	Percentage
Age group		
<18 years	12	1.4%
18–23 years	239	28.3%
24–30 years	285	33.7%
31–35 years	132	15.6%
36–40 years	85	10.1%
41–45 years	41	4.9%
>45 years	51	6.0%
Residence		
Urban	211	25.0%
Rural	634	75.0%
Ethnicity		
Amhara	829	98.1%
Oromo	14	1.7%
Tigre	2	0.2%
Religion		
Orthodox	727	86.0%
Protestant	41	4.9%
Muslim	74	8.8%
Others	3	0.4%
Current marital status		
Single	275	32.5%
Married	470	55.6%
Widowed	37	4.4%
Divorced	63	7.5%
Educational status		
Cannot read and write	121	14.3%
Can read and write	224	26.5%
Primary	107	12.7%
Secondary	261	30.9%
College and above	132	15.6%
Occupation		
Self-employed	215	25.4%
House wife	296	35.0%
Private employee	94	11.1%
Government employee	85	10.1%
Others (student)	155	18.3%
currently living with		
With husband/friend	427	50.5%
With extended family	236	27.9%
With kids	44	5.2%
Alone	112	13.3%
Others	26	3.1%
Family number		
≤2	268	31.7%
3–5	455	53.8%
≥6	122	14.4%
Monthly income		
Less than 36 USD	296	35.0%
36–71.982 USD	238	28.2%
72–180 USD	223	26.4%
Greater than 180 USD	88	10.4%

NB: Religion other = Catholic, Occupation other = Student, living with other = Friends.

### Sexual history of participants

According to the findings of this study, (688, 81.4%) of the respondents reported ever having engaged in sexual intercourse. Among them, (416, 60.5%) had their first sexual experience after the age of 18, and (366, 53.2%) women had their first sexual experience within the context of marriage. Approximately one-fifth of the women, (134, 19.5%), had more than one sexual partner, while (493, 58.3%) of the participants reported having a constant sexual partner.

Furthermore, (659, 78.0%) of the participants had heard about Sexual and Reproductive Health (SRH), with mass media serving as the primary source of information for (324, 48.9%) of the respondents. Likewise, (232, 27.5%) women had previous exposure to GBV (GBV), and (155, 18.3%) of them had a history of experiencing sexual violence.

### Obstetric/gynecologic history

This study revealed that more than half of the women, (453, 53.6%), had been pregnant at some point, and 119 (26.3%) had experienced abortion. The majority of the women, (479, 56.7%), had given birth to more than two children, and (443, 52.4%) of them had two or more children (Table 2).

**Table 2:** Previous fertility characteristics of women residing in war-affected woredas, in North Shewa zone, Amhara, Ethiopia, 2022.

Variables	Count	Percentage
Ever been pregnant		
Yes	453	53.6%
No	392	46.4%
Ever have an unwanted pregnancy		
Yes	103	22.7%
No	350	77.3%
Ever had abortion		
Yes	119	26.3%
No	334	73.7%
Ever had an induced abortion		
Yes	28	6.2%
No	425	93.8%
Number of alive children		
No child	98	11.6%
≤2 child	304	36.0%
>2 child	443	52.4%
Parity of a woman		
Null para	73	8.6%
≤2	293	34.7%
>2	479	56.7%

### Prevalence of GBV

Based on the results of this research, the overall prevalence of GBV in the study location was (490, 58%). Among these cases, (254, 30.1%) encountered physical violence, (466, 55%) faced psychological violence, and (135, 16%) disclosed incidents of sexual violence.

**Figure 1:**
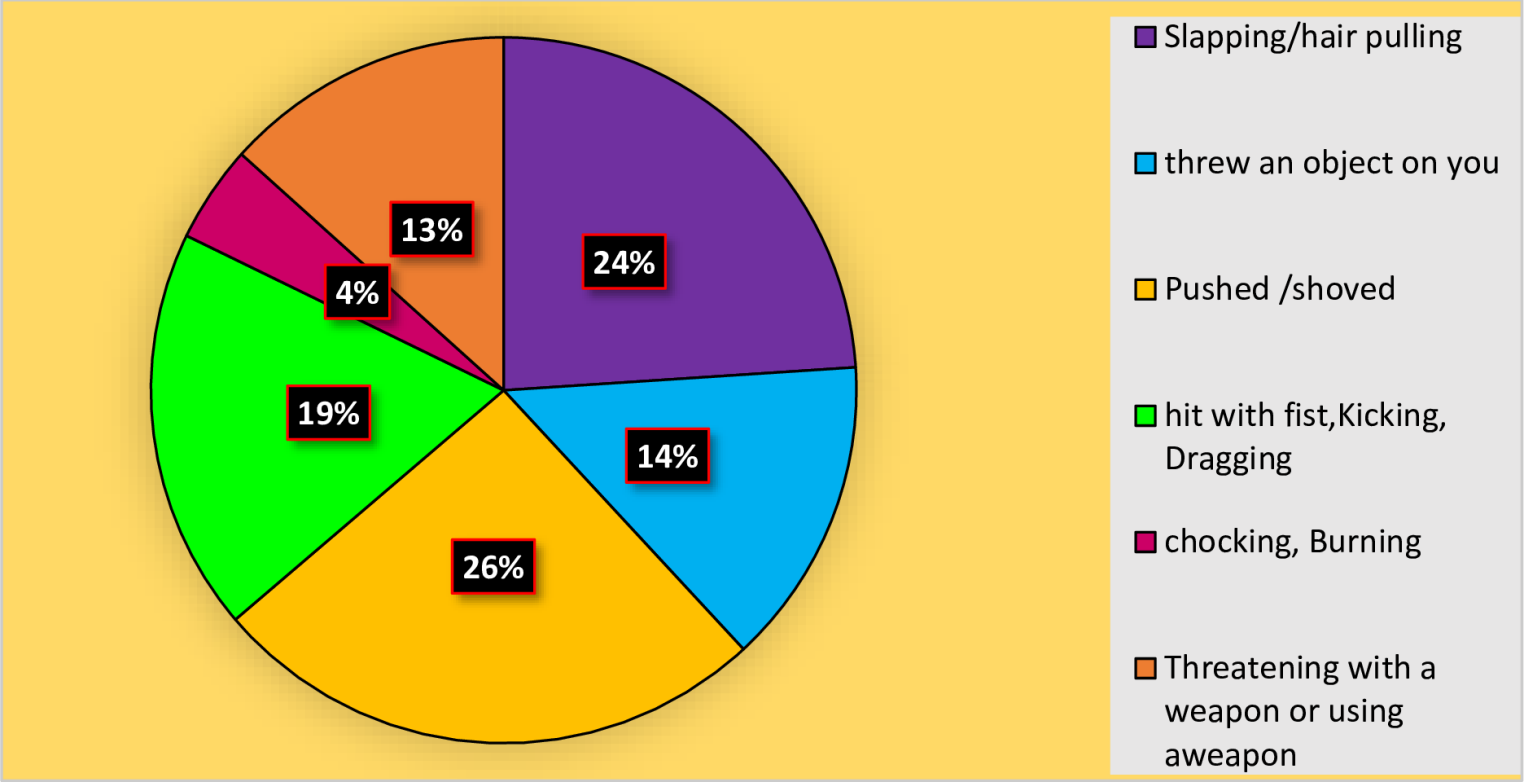
Types of physical violence faced by participants residing in war-affected woredas, in the North Shewa zone, Amhara, Ethiopia, 2022.

Regarding physical violence, among women who faced it, (66, 26%) were subjected to pushing or shoving, (61, 24%) experienced slapping or hair pulling, and (48, 19%) were Survivors of being hit with fists, kicking, or dragging (Figure 1).

**Figure 2:**
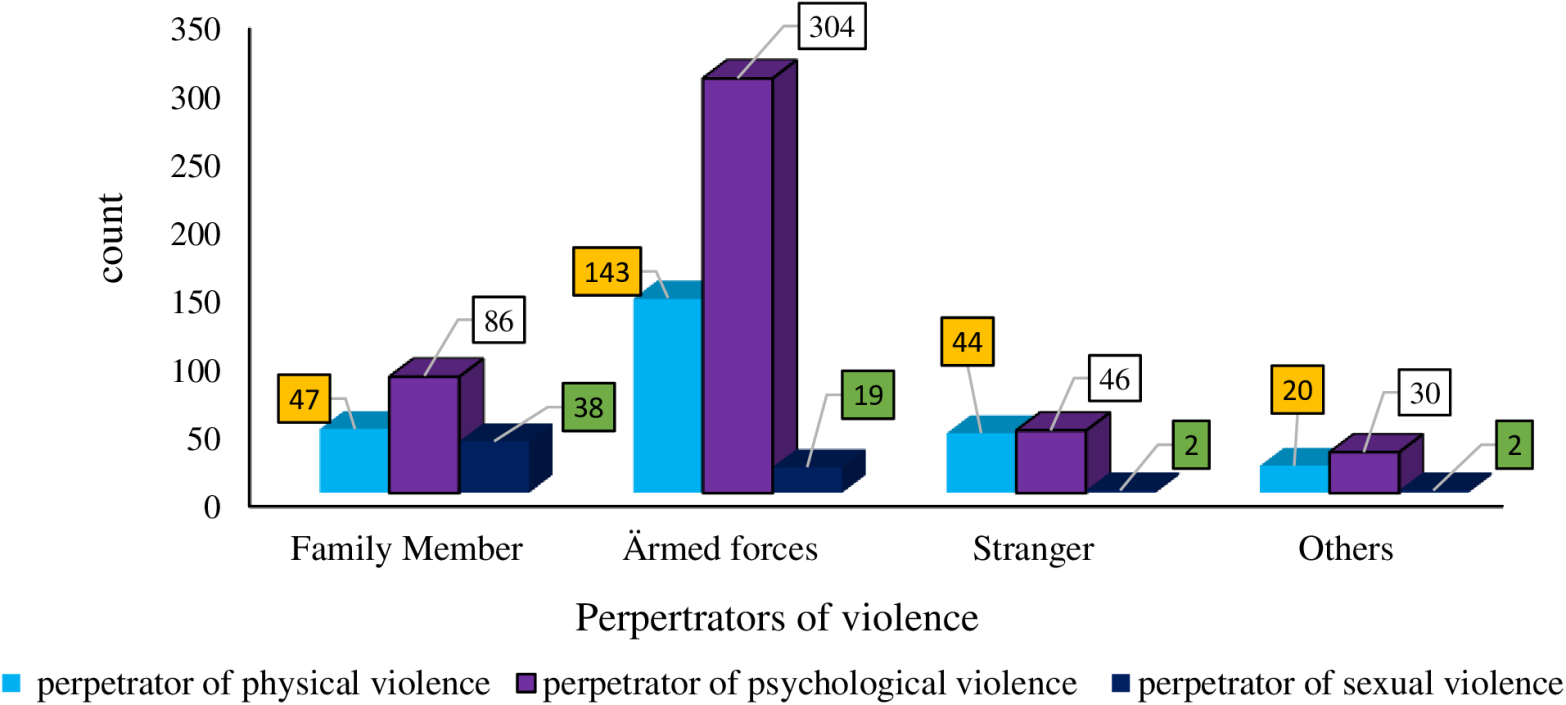
Perpetrators of gender-based violence in war-affected woredas, North Shewa zone, Amhara, Ethiopia, 2022.

A majority of the physical violence, (161, 63.4%), occurred during the conflict period, with (143, 56.3%) of these cases involving armed forces, and (161, 63.4%) of the women experiencing physical violence within their homes. Notably, only a quarter of the women, (68, 26.8%), reported their physical violence, while (36, 51.4%) confided in family members, close friends, and neighbors. The most commonly reported reason for not reporting the violence was fear of criminals, with (113, 56.8%) of the respondents citing this, and (38, 15.0%) experienced excessive bleeding, while (17, 6.6%) suffered the loss of a pregnancy as severe complications resulting from physical violence (Table 3 and Figure 2).

**Table 3:** Characteristics of physical violence faced by participants residing in war-affected woredas, in the North Shewa zone, Amhara, Ethiopia, 2022.

Characteristics of physical violence	Count	Percentage
Perpetrator physical violence		
Family member	47	18.5%
Armed forces	143	56.3%
Stranger	44	17.3%
Others	20	7.9%
Place of faced physical violence		
At home	161	63.4%
Public area during the conflict	77	30.3%
Others	16	6.3%
Report physical violence		
Yes	68	26.8%
No	186	73.2%
Violence reported to		
Police	22	31.4%
Gender office	12	17.1%
Others (family members, close friends, and neighborhoods)	36	51.4%
Reason for not reporting it		
Legal body not helpful	29	14.6%
Afraid of the criminals	113	56.8%
Ï don't know	33	16.6%
Others (legal services were not available)	24	12.1%
Challenges faced due to physical violence		
Bone fracture	21	8.3%
Too much bleeding	38	15.0%
Loss of pregnancy	17	6.6%
Headache	5	2.0%
Minor problems but I forget it	96	37.8%
Others (physical injuries, fractures)	77	30.3%

In a similar vein, this study indicated that (178, 38.2%) individuals faced humiliation, (184, 39.5%) experienced intimidation or relegation, and (119, 25.5%) encountered spiting, while (102, 21.9%) felt belittled.

**Figure 3:**
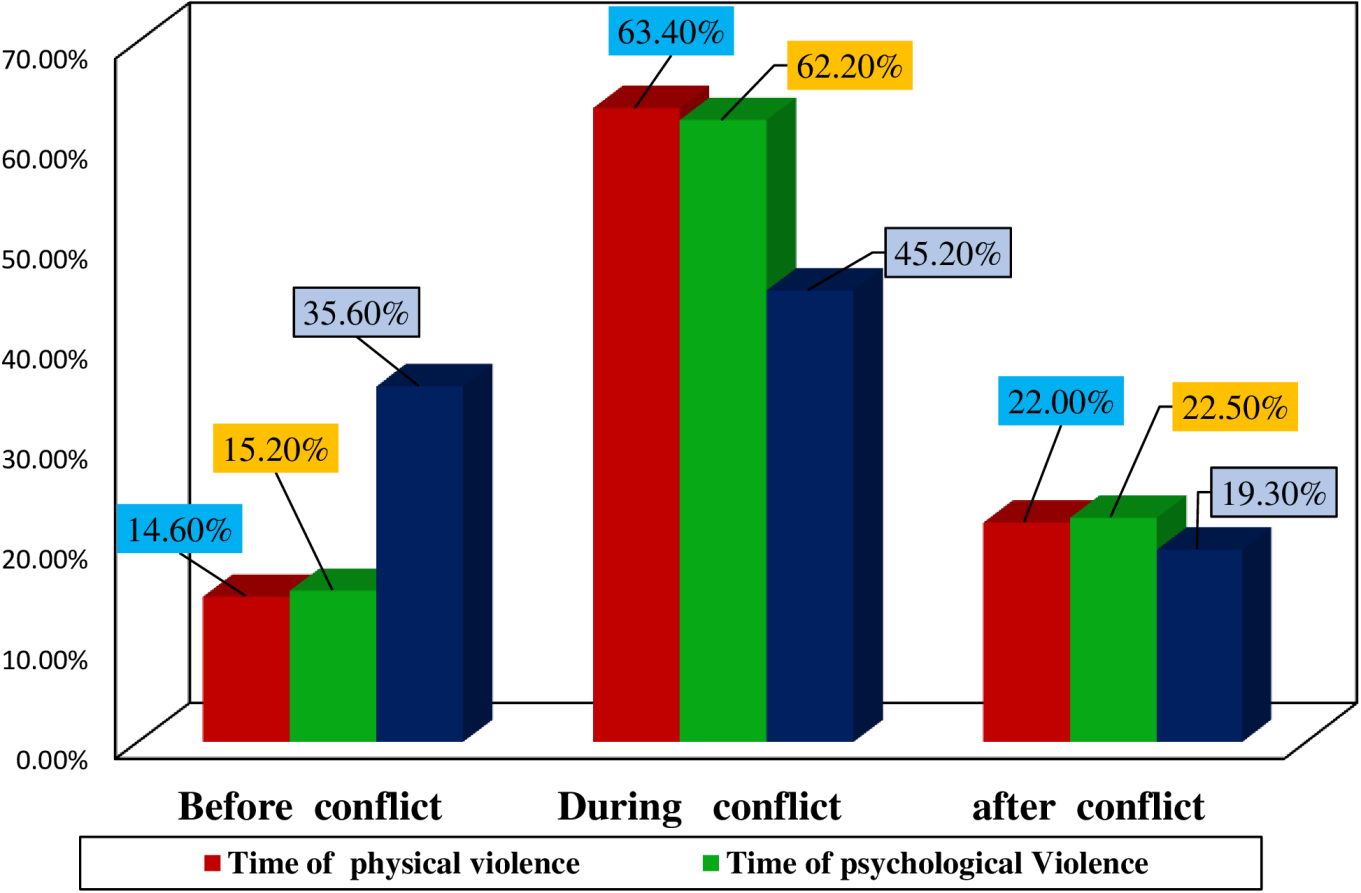
Timing of occurrence of gender based violence of participants residing in war-affected woredas, in North Shewa zone, Amhara, Ethiopia, 2022.

The majority of instances of psychological violence, (290, 62.2%), took place during the conflict period, with (304, 65.2%) of these incidents involving armed forces, and (254, 54.5%) of the Survivors enduring psychological violence within their homes. Moreover, only one-fifth of the women, (93, 20.0%), reported their experiences of psychological violence, while (45, 46.4%) confided in family members, close friends, and neighbors. Approximately half of the survivors, (179, 46.6%), chose not to report their psychological violence due to their fear of the perpetrators (Table 4 and Figure 3).

**Table 4:** Characteristics of psychological violence faced by participants residing in war-affected woredas, in the North Shewa zone, Amhara, Ethiopia, 2022.

Characteristics of psychological violence	Count	Percentage
Perpetrator of psychological violence		
Family member	86	18.5%
Armed forces	304	65.2%
Stranger	46	9.9%
Others	30	6.4%
Place of faced psychological violence		
My home	254	54.5%
Public area during the conflict	183	39.3%
Others (school and work area)	29	6.2%
Report psychological violence		
Yes	93	20.0%
No	373	80.0%
Psychological violence reported to		
Police	43	44.3%
Gender office	9	9.3%
Others (family members, close friends, and neighborhoods)	45	46.4%
Reason for not reporting it		
Legal body not helpful	90	23.4%
Afraid of the criminals	179	46.6%
Ï don't know	82	21.4%
Others (legal services were not available)	33	8.6%

The results of this study also revealed that (56, 28.7%) respondents experienced unwanted touching of the breast or buttock, (54, 27.7%) faced attempted forced sexual acts, (51, 26.2%) were subjected to unwanted kissing, (22, 11.3%) were coerced into watching explicit sexual content, and (12, 6.2%) reported incidents of rape.

Furthermore, a majority of sexual violence, (85, 63.0%), occurred in the Survivors' own homes, with only (25, 18.5%) of them reporting these incidents. Interestingly, more than half of the Survivors (52.0%) chose to report the sexual violence to the police. The main reason for not reporting these incidents was the fear of the perpetrators, as noted by (47, 39.8%) respondents. Additionally, (61, 45.2%) of them experienced complete rape, of which (19, 31.1%) were reported, and (14, 23%) of the incidents involved two or more perpetrators. A total of (31, 50.8%) survivors received healthcare services (Table 5).

**Table 5:** Characteristics of sexual violence faced by participants residing in war-affected woredas, in the North Shewa zone, Amhara, Ethiopia, 2022.

Characteristics of sexual violence	Count	Percent
Place of faced sexual violence		
My home	85	63.0%
Public area during the conflict	31	23.0%
Others (school, workplace)	19	14.1%
Report sexual violence		
Police	13	52.0%
Gender office	8	32.0%
Others (family members, friends, neighbors)	4	16.0%
Reason for not reporting it		
Legal body not helpful	36	30.5%
Afraid of the criminals	47	39.8%
Ï don't know	23	19.5%
Others (legal services were not available)	12	10.2%
Timing of sexual violence		
Before the conflict	20	32.8%
During the conflict	29	47.5%
After the conflict	12	19.7%
Place of faced rape		
My home	30	49.2%
Public area during the conflict	18	29.5%
Others (school, work area)	13	21.3%
Rape reported to		
Police	14	73.7%
Family members	3	15.8%
Others (family members, friends, neighbors)	2	10.5%
Reason for not reporting the rape		
Feeling shame/embarrass	13	27.7%
Fear of being stigmatized	9	19.1%
Fear of rejection by family/community	7	14.9%
Did not trust anyone	8	17.0%
Thought nothing could be done	4	8.5%
Fear of more violence if perpetrators hear	4	8.5%
I don't know	2	4.3%
Frequency of experienced complete rape		
One	39	63.9%
Two	16	26.2%
Three or more	6	9.8%
Perpetrator of the rape		
Boyfriend	32	52.5%
Husband	6	9.8%
Stranger	2	3.3%
Armed force	19	31.1%
Other	2	3.3%
Get health services after the rape		
Yes	33	54.1%
No	28	45.9%
Reason for not seeking medical care		
Did not need medical care	7	23.3%
Did not know where to go	6	20.0%
Medical care not available	5	16.7%
Embarrassment	2	6.7%
Afraid of further violence	2	6.7%
Had no money/Transport	2	6.7%
Being wartime	6	20.0%
Strategies used by the perpetrator		
Made me drunk	7	11.5%
The threat of harm/intimidation	39	63.9%
Forced me to take a drug	11	18.0%
Other (specify)	4	6.6%
Challenges faced after been raped		
Amenorrhea with pregnancy	6	9.8%
Abortion	15	24.6%
Urination without knowing	4	6.6%
Vaginal bleeding	16	26.2%
Bloody stools	3	4.9%
HIV infection	2	3.3%
Psychological problems	15	24.6%

NB: perpetrator “Others” = teachers, family members.

Similarly, among women survivors of sexual violence, nearly half (61, 45.2%) of them had experienced complete rape, and about half (30, 49.2%) of these incidents occurred in their own homes. Out of these women, (19, 31.1%) reported the incidents of rape, and (14, 73.7%) reported them to the police. The most common reason for not reporting these cases was a feeling of shame or embarrassment (13, 27.7%) as indicated in (Table 5).

Concerning the frequency of complete rape, (39, 63.9%) women experienced it once, while (16, 26.2%) faced rape on two or more occasions. Of the rape survivors, (32, 52.5%) were assaulted by their boyfriends, and (19, 31.1%) reported incidents involving armed forces. The majority of the perpetrators (39, 63.9%) employed the threat of harm or intimidation as a strategy to assault the survivors.

Additionally, nearly half of the Survivors (28, 45.9%) did not receive healthcare services after the rape, with (6, 20.0%) of them citing lack of awareness of where to seek help and the wartime conditions as the primary reasons for not accessing healthcare facilities. The most common complications faced by the women after experiencing rape were vaginal bleeding (16, 26.2%) and abortion (15, 24.6%) (Table 5).

### Consequences of GBV

**Figure 4:**
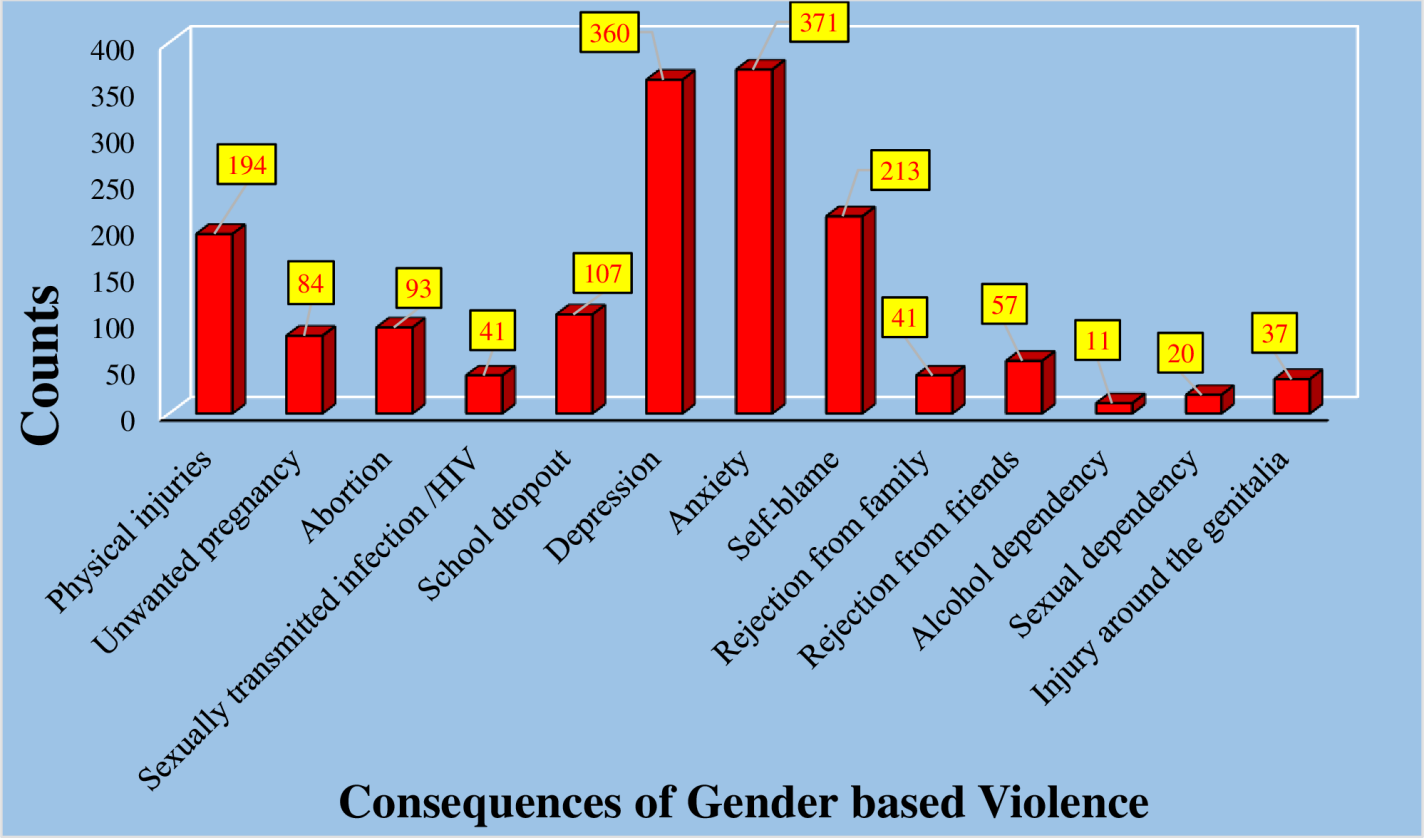
Health consequences of gender-based violence among women residing in war-affected woredas, North Shewa, Amhara, 2022.

Consequently, women living in war-affected woredas of the North Shewa zone suffered various health repercussions as a result of experiencing GBV either personally or witnessing it in their vicinity. Specifically, (371, 43.9%) experienced anxiety, (360, 42.6%) dealt with depression, (194, 23.0%) sustained physical injuries, (213, 25.2%) grappled with self-blame, (107, 12.7%) had to drop out of school, and (93, 11.0%) respondents went through abortion (Figure 4).

### Factors associated with GBV

The findings of this study revealed that residing in an urban area increased the odds of experiencing GBV by more than two and a half times AOR = 2.66, CI, (1.82–3.89). On the other hand, educational status of Secondary education AOR = 0.33, CI, (0.19–0.57), and College and above AOR = 0.17, CI, (0.09–0.35) decreased the odds of GBV by 67%, and 83%, respectively, compared to women who could not read and write.

The multiple variable logistic regression analysis output also indicated that certain occupational statuses increased the odds of GBV. Housewife AOR = 1.88, CI, (1.20–2.94), Private employees AOR = 6.95, CI, (3.70–13.04), Government employees AOR = 5.80, CI, (2.92–11.50), and Others (Students) AOR = 3.46, CI, (1.98–6.01) increased the odds by about 2, 7, 6, and three and a half times, respectively, compared to those who were self-employed.

Furthermore, women who had never engaged in sexual intercourse were 54% less likely to experience GBV AOR = 0.46, CI, (0.25–0.83) compared to women with a history of sexual activity. Similarly, women who had heard about SRH were 41% less likely to face GBV AOR = 0.59, CI, (0.40–0.89) than those who had not.

Additionally, women who had previously been exposed to GBV AOR = 0.24, CI, (0.15–0.38) and women with a previous history of sexual violence AOR = 0.30, CI, (0.16–0.57) were 76% and 70% less likely, respectively, to be Survivors of GBV compared to their counterparts.

Lastly, this study also revealed that women with two or more sexual partners increased the odds of GBV by about two times AOR = 1.84, CI, (1.13–2.99) compared to those with only one sexual partner (Table 6).

**Table 6:** Factors associated with gender-based violence among women residing in war-affected woredas in North Shewa, Amhara, Ethiopia, 2022.

	Gender-based violence		COR (95% CI)	AOR (95% CI)
	No	Yes		
	Count (%)	Count/%		
Residence				
Rural	114 (32.1%)	97/19.8%	1	1
Urban	241 (67.9%)	393/80.2%	1.92 (1.40–2.63)	**2.658** **(1.82–3.89)**
Current marital status				
Single	128 (36.1%)	147/30.0%	1	1
Married	185 (52.1%)	285/58.2%	1.34 (0.99–1.81)	1.75 (1.09–2.82)
Widowed	18 (5.1%)	19/3.9%	0.92 (0.46–1.83)	1.34 (0.54–3.32)
Divorced	24 (6.8%)	39/8.0%	1.42 (0.8–2.48)	1.15 (0.56–2.40)
Educational status				
Can’t read & write	45 (2.7%)	76/15.5%	1	1
Can read & write	66 (18.6%)	158/32.2%	1.42 (0.89–2.26)	0.98 (0.58–1.67)
Primary	48 (13.5%)	59/12.0%	0.73 (0.43–1.24)	0.58 (0.31–1.08)
Secondary	125 (35.2%)	136/27.8%	0.64 (0.41–1.00)	**0.33** **(0.19–0.57)**
College and above	71 (20.0%)	61/12.4%	0.51 (0.31–0.84)	**0.17** **(0.09–0.35)**
Occupation				
Self-employed	117 (33.0%)	98/20.0%	1	1
Housewife	118 (33.2%)	178/36.3%	1.80 (1.26–2.57)	**1.88** **(1.20–2.94)**
Private employee	28 (7.9%)	66/13.5%	2.81 (1.68–4.72)	**6.95** **(3.70–13.04)**
Gov’t employee	33 (9.3%)	52/10.6%	1.88 (1.13–3.14)	**5.80** **(2.92–11.50)**
Others (students)	59 (16.6%)	96/19.6%	1.94 (1.28–2.96)	**3.46** **(1.98–6.01)**
Ever had sexual intercourse				
Yes	269 (75.8%)	409/83.5%	1	1
No	86 (24.2%)	81/16.5%	0.73 (0.51–1.03)	**0.46** **(0.25–0.83)**
Heard about SRH				
No	228 (64.2%)	390/79.6%	1	1
Yes	127 (35.8%)	100/20.4%	0.80 (0.58–1.11)	**0.59** **(0.40–0.89)**
Had previous GBV exposure				
Yes	37 (10.4%)	195/39.8%	1	1
No	318 (89.6%)	295/60.2%	0.18 (0.12-.26)	**0.24** **(0.15–0.38)**
History of sexual violence				
Yes	20 (5.6%)	135/27.6%	1	1
No	335 (94.4%)	355/72.4%	**0.16** **(0.10-.26)**	**0.30** **(0.16–0.57)**
Ever been pregnant				
Yes	180 (50.7%)	273/55.7%	1	1
No	175 (49.3%)	217/44.3%	0.82 (0.62–1.08	1.08 (0.70–1.67)
Number of household members				
≤2 members	109 (30.7%)	159/32.4%	1	1
3–5 members	198 (55.8%)	257/52.4%	0.89 (0.66–1.21	0.75 (0.52–1.09)
≥6 members	48 (13.5%)	74/15.1%	1.06 (0.68–1.64)	0.66 (0.39–1.14)
Number of sexual partners				
1 partner	243 (68.5%)	311/63.5%	1	1
2 and more	112 (31.5%)	179/36.5%	1.79 (1.30–2.46)	**1.84** **(1.13–2.99)**
Monthly income				
less than 2,000 ETB	81 (22.8%)	116/23.7%	1	1
2,000–3,999 ETB	126 (35.5%)	136/27.8%	0.75 (0.52–1.09)	0.83 (0.52–1.32)
4,000–10,000 ETB	128 (36.1%)	211/43.1%	0.98 (0.68–1.41)	1.12 (0.70–1.79)
>10,000 ETB	20 (5.6%)	27/5.5%	1.86 (1.08–3.22)	1.12 (0.50–2.49)

## Discussion

Our study revealed a high prevalence of GBV, where 58.0% experienced psychological, physical and/or sexual violence, during conflict in North Shewa, Amhara, Ethiopia.

So the results of this study indicate a higher prevalence of GBV. As GBV prevalence data is highly problematic as the methods used to collect data are varied, self-reporting is influenced by culture, fear, trauma, repeat victimization, and it is an understudied subject. In general studies conducted among conflict affected areas such as Syrian women in Lebanon Liberia Zimbabwe and South Sudan had similar data with increase in conflict associated GBV (30.8%), (81.6%) (95%, 31%, 92% & 65% experienced faced physical violence, rape by a stranger, encountered spousal rape, and were Survivors of forced marriages), (65%) respectively (15–18). This is due to; Conflict or war situations often exacerbate gender-based violence (GBV), leading to increased risks and vulnerabilities for women and girls. In times of conflict, existing inequalities and power imbalances are magnified, making women more susceptible to various forms of violence, including sexual assault, domestic abuse, and exploitation. Furthermore, displacement, breakdown of social structures, and limited access to resources can further intensify the prevalence of GBV (19). Therefore, addressing GBV in conflict situations requires comprehensive strategies that encompass prevention, protection, and support services for survivors. Preventive measures that ought to be in place including prioritization of GBV prevention and services by humanitarian agencies, government, and UN including training of men and armed groups to respect the laws of the country.

Additionally, this study found that residing in urban areas during the conflict increased the likelihood of experiencing GBV compared to rural areas. This observation aligns with research indicating the impact of urbanization on GBV in the global south (20). This trend may be attributed to armed forces often favoring cities and urban areas for control, positioning governmental institutions at the heart of the conflict.

Furthermore, we observed that having a higher level of education served as a protective factor against gender-based violence, in line with findings from related studies exploring the socio-demographic predictors of GBV (21, 22). Increased educational attainment among women appeared to enhance their awareness of Sexual and Reproductive Health (SRH) information, enhance their socioeconomic status by broadening job opportunities, and promote financial independence, all of which are vital in preventing various forms of violence (22, 23). Conversely, low academic performance might hinder their access to critical information about SRH, their roles within relationships, and strategies to protect themselves from such forms of violence.

In this study, it was observed that individuals who had ever experienced sexual exposure and those who had not received information about Sexual and Reproductive Health (SRH) were more likely to face an increased risk of gender-based violence. This correlation aligns with findings from studies conducted in Gonder, Wolaita, Bahir Dar, and Dessie City (24–27). This connection may be attributed to the fact that engaging in conversations about sexual and reproductive health and having access to such information can enhance life skills and decision-making abilities. Moreover, it enables women to better comprehend potential risks and complications in advance.

Similarly, a history of previous GBV (GBV) and sexual violence was found to be a significant factor increasing the risk of GBV in this study area. This is likely because the underlying issues that previously made women vulnerable to violence have not been addressed, leaving them exposed to recurring instances of violence (28).

Furthermore, this study established that having more than one sexual partner was a statistically significant factor associated with GBV (GBV) in this study area. This discovery aligns with research conducted in Gonder, which reported that multiple sexual partnerships or unions increased the risk of GBV (24). This correlation may be attributed to the fact that having multiple sexual partners can heighten vulnerability to intimate partner violence involving multiple perpetrators.

In conclusion, this study identified that women residing in war-affected woredas of the North Shewa zone faced a multitude of health consequences resulting from Gender-Based Violence, whether directed at them or occurring nearby. The most frequently reported consequences of GBV in this study area included anxiety, depression, physical injuries, self-blame, school dropouts, and induced abortions. This finding is consistent with various studies highlighting that women who experience GBV are at risk of developing numerous health consequences affecting their physical, mental, and reproductive well-being (29–31). This could be attributed to perpetrators often using physical force to assault their Survivors, who may be caught unprepared, leading to undesired pregnancies, sexually transmitted infections (STIs), and related psychological and physical complications.

Our findings should be viewed in light of both the strengths and limitations of our study. Notably, population-based studies on GBV are scarce, highlighting a strength of our research, along with the short recall period. However, we acknowledge certain limitations. We did not engage healthcare workers to assess available services for survivors, and we did not gather data on the measures taken by humanitarian agencies, government bodies, and military commanders to prevent GBV and safeguard civilians.

## Conclusion

In summary, our study identified a higher prevalence of GBV (GBV) in our study area, with 490 (58%) individuals affected. Among them, 254 (30.1%) experienced physical violence, 466 (55%) were subjected to psychological violence, and 135 (16%) endured sexual violence. This prevalence exceeded that reported in other studies conducted in different parts of Ethiopia and the World Health Organization's 2018 estimates for Violence Against Women (VAW), which was 31%.

Moreover, our analysis revealed that residence, educational status, occupation, sexual experience, awareness of Sexual and Reproductive Health (SRH), prior exposure to GBV, history of sexual violence, and the number of sexual partners were significant factors associated with GBV in our study area. Conversely, current marital status, a history of being pregnant, the number of household members, and monthly income did not exhibit a statistically significant association with GBV, although they had been significant predictors of GBV in other studies.

Lastly, our study shed light on the multiple mental health consequences accompanying GBV, including anxiety, depression, self-blame, physical injuries, school dropouts, and induced abortions.

### Recommendations

#### To debre berhan university

Collaborate with health facilities and other stakeholders to Provide and support Sexual and Reproductive Health (SRH) and psychosocial services in affected woredas, and establish and support rehabilitation centers for Survivors of GBV (GBV) in war-affected districts.

#### To the North Shewa zone health department

Enhance access to SRH information through various modalities, Work on improving women's status, and promote and support services for Survivors of GBV.

#### To researchers

Conduct further investigations using advanced study designs, including qualitative data analysis.

#### Strength of the study

The study was community-based and aimed to assess not only the prevalence of GBV but also its associated health consequences, it was conducted shortly after the conflict, reducing the potential for recall bias, and psychological support and linkage/referral of Survivors were provided.

#### Limitations of the study

The study relied on participants' self-reporting to identify and diagnose GBV and its health consequences. Additionally, We did not engage healthcare workers to assess available services for survivors, and we did not gather data on the measures taken by humanitarian agencies, government bodies, and military commanders to prevent GBV and safeguard civilians.

## Data Availability

The raw data supporting the conclusions of this article will be made available by the authors, without undue reservation.

## References

[1] Degener T Koster-Dreese Y. Declaration on the elimination of violence against women: by general assembly resolution 48/104 of 20 December 1993. In: Degener T Koster-Dreese Y, editors. Human Rights and Disabled Persons. Leiden: Brill Nijhoff (1995). p. 416–22.

[2] Organization WH. Violence against women: intimate partner and sexual violence against women: evidence brief. World Health Organization (2019).

[3] Refugees UNHCf. Sexual and Gender-Based Violence Against Refugees, Returnees, and Internally Displaced Persons. Geneva: UNHCR (2003).

[4] Vu A Adam A Wirtz A Pham K Rubenstein L Glass N The prevalence of sexual violence among female refugees in complex humanitarian emergencies: a systematic review and meta-analysis. PLoS Curr. (2014) 6. 10.1371/currents.dis.835f10778fd80ae031aac12d3b533ca7 24818066 PMC4012695

[5] Cohen DK Green AH Wood EJ. Wartime Sexual Violence: Misconceptions, Implications, and Ways Forward. Washington, DC: Institute of Peace (2013).

[6] Ward J Vann B. Gender-based violence in refugee settings. Lancet. (2002) 360:s13–4. 10.1016/S0140-6736(02)11802-2 12504485

[7] Organization WH. Devastatingly pervasive: 1 in 3 women globally experience violence. News (2021).

[8] Ellsberg M Heise L, Organization WH. Researching Violence Against Women: A Practical Guide for Researchers and Activists. Geneva: World Health Organization (WHO) (2005).

[9] OCHA. According to Ethiopia national displacement report 10 by IOM, 2.11 million IDPs were tracked through the emergency site assessment (ESA) round 8 which is a monthly tool used to track internal displacement in Tigray, Afar and Amhara regions caused by the Northern Ethiopia Crisis (2021).

[10] FDRE. Fifth national report on progress made in the implementation of the Beijing declaration and platform for action (Beijing +25) (2019).

[11] Plaut M Vaughan S. Understanding Ethiopia’s Tigray War. London: Hurst Publishers (2023).

[12] Abdulkadr AA Neszmelyi GI. Root causes and the socio-economic impact of the ongoing war between the TPLF and the federal government of Ethiopia (2 November 2020–15 October 2021). Hungarian J Afr Stud. (2021) 15:37.

[13] Abbink G. The Politics of Conflict in Northern Ethiopia, 2020–2021: a Study of War-Making, Media Bias and Policy Struggle. African Studies Centre Leiden: The Netherlands, ASCL Working Paper (2021). p. 152.

[14] Yigzaw T Yibric A Kebede Y. Domestic violence around gondar in northwest Ethiopia. Ethiop J Health Dev. (2004) 18(3):133–9.

[15] Reese Masterson A Usta J Gupta J Ettinger AS. Assessment of reproductive health and violence against women among displaced Syrians in Lebanon. BMC Women’s Health. (2014) 14(1):1–8. 10.1186/1472-6874-14-25 24552142 PMC3929551

[16] Omanyondo M. WHO sexual gender-based violence and health facility needs assessment (Lofa Nimba, Grand Gedeh and Grand Bassa Counties) (2005). Available online at: https://www.who.int/hac/crises/lbr.Liberia_GBV_2004_FINAL.pdf (Accessed December 18, 2022).

[17] Mukanangana F Moyo S Zvoushe A Rusinga O. Gender based violence and its effects on women’s reproductive health: the case of Hatcliffe, Harare, Zimbabwe. Afr J Reprod Health. (2014) 18(1):110–22. 24796175

[18] Cluster GH. Gender-based violence in health emergencies. April.

[19] Human Rights Watch. World report 2018: events of 2017. (2018). Available online at: https://www.hrw.org/world-report/2018 (Accessed December 18, 2022).

[20] McIlwaine C. Urbanization and gender-based violence: exploring the paradoxes in the global South. Environ Urban. (2013) 25(1):65–79. 10.1177/0956247813477359

[21] Peraica T Kovačić Petrović Z Barić Ž Galić R Kozarić-Kovačić D. Gender differences among domestic violence help-seekers: socio-demographic characteristics, types and duration of violence, perpetrators, and interventions. J Fam Violence. (2021) 36:429–42. 10.1007/s10896-020-00207-8

[22] Erten B Keskin P. For better or for worse? Education and the prevalence of domestic violence in Turkey. Am Econ J-Appl Econ. (2018) 10(1):64–105. 10.1257/app.20160278

[23] El-Gamal SMA Dawood AS Elbohoty SB Elkest HRA. Effect of educational intervention and female empowerment in facing intimate partner violence: quasi-experimental study. Int J Nurs. (2022) 9(1):1–12. 10.15640/ijn.v9n1a1

[24] Muche MAA. Magnitude and correlates of gender-based violence among married women in northwest Ethiopia. Afr J Med Med Sci. (2017) 46(2):213–25.

[25] Tantu T Wolka S Gunta M Teshome M Mohammed H Duko B. Prevalence and determinants of gender-based violence among high school female students in Wolaita Sodo, Ethiopia: an institutionally based cross-sectional study. BMC Public Health. (2020) 20(1):1–9. 10.1186/s12889-020-08593-w 32316941 PMC7345512

[26] Belay HG Liyeh TM Tassew HA Ayalew AB Goshu YA Mihretie GN. Magnitude of gender-based violence and its associated factors among female night students in Bahir Dar City, Amhara region, Ethiopia. Int J Reprod Med. (2021) 2021:1–7. 10.1155/2021/6694890 PMC8057901 33954167

[27] Gebrie S Wasihun Y Abegaz Z Kebede N. Gender-based violence and associated factors among private college female students in Dessie City, Ethiopia: mixed method study. BMC Women’s Health. (2022) 22(1):513. 10.1186/s12905-022-02076-3 36503440 PMC9743651

[28] Wanjiru Q. Causes and effects of gender-based violence. A critical literature review. J Gend Relat Stud. (2021) 2(1):43–53. 10.47941/jgrs.742

[29] Cluster GH. Gender-based violence in health emergencies. (2020):2–38.

[30] García-Moreno C Jansen HA Ellsberg M Heise L Watts C. WHO Multi-Country Study on Women’s Health and Domestic Violence Against Women. Geneva: World Health Organization (WHO) (2005).

[31] Heise L Ellsberg M Gottmoeller M. A global overview of gender-based violence. Int J Gynaecol Obstet. (2002) 78:S5–14. 10.1016/S0020-7292(02)00038-3 12429433

